# Circulating Extracellular Vesicles Are Associated with Disease Severity and Interleukin-6 Levels in COPD: A Pilot Study

**DOI:** 10.3390/jcm10215014

**Published:** 2021-10-28

**Authors:** Dario Nieri, Marta Daniele, Stefania Lombardi, Erica Bazzan, Sabrina Santerini, Giovanna De Cusatis, Barbara Vagaggini, Manuel G. Cosio, Marina Saetta, Pierluigi Paggiaro, Alessandro Celi, Tommaso Neri

**Affiliations:** 1 Sezione a Valenza Dipartimentale di Fisiopatologia Respiratoria e Riabilitazione Respiratoria Universitaria, Dipartimento Cardio-Toraco Vascolare, Azienda Ospedaliero Universitaria Pisana, 56124 Pisa, Italy; marta.daniele91@gmail.com (M.D.); s.santerini@ao-pisa.toscana.it (S.S.); giannadecusatis@hotmail.it (G.D.C.); b.vagaggini@ao-pisa.toscana.it (B.V.); pierluigi.paggiaro@unipi.it (P.P.); 2 Centro Dipartimentale di Biologia Cellulare Cardiorespiratoria, Dipartimento di Patologia Chirurgica, Medica, Molecolare e dell’Area Critica, Università degli Studi di Pisa, 56124 Pisa, Italy; alessandro.celi@unipi.it (A.C.); tommaso.neri79@for.unipi.it (T.N.); 3 SSD Analisi ChimicoCliniche ed ImmunoAllergologia, USL Toscana Nordovest, 54100 Massa, Italy; stefania.lombardi@uslnordovest.toscana.it; 4 Dipartimento di Scienze Cardio-Toraco-Vascolari e Sanità Pubblica, Università degli Studi di Padova, 35122 Padova, Italy; erica.bazzan@unipd.it (E.B.); manuel.cosio@mcgill.ca (M.G.C.); marina.saetta@unipd.it (M.S.)

**Keywords:** chronic obstructive pulmonary disease, extracellular vesicles, systemic inflammation, endothelium

## Abstract

Chronic obstructive pulmonary disease (COPD) is a complex condition in which systemic inflammation plays a role in extrapulmonary manifestations, including cardiovascular diseases: interleukin (IL)-6 has a role in both COPD and atherogenesis. The 2011 GOLD document classified patients according to FEV1, symptoms, and exacerbations history, creating four groups, from A (less symptoms/low risk) to D (more symptoms/high risk). Extracellular vesicles (EV) represent potential markers in COPD: nevertheless, no studies have explored their value in association to both disease severity and inflammation. We conducted a pilot study to analyze circulating endothelial-(E) and monocyte-derived (M) EV levels in 35 COPD patients, who were grouped according to the 2011 GOLD document; the relationship between EV and plasmatic markers of inflammation was analyzed. We found a statistically significant trend for increasing EEV, MEV, IL-6, from group A to D, and a significant correlation between EEV and IL-6. The associations between both EEV and MEV and disease severity, and between EEV and IL-6, suggest a significant interplay between pulmonary disease and inflammation, with non-respiratory cells (endothelial cells and monocytes) involvement, along with the progression of the disease. Thus, EV might help identify a high-risk population for extrapulmonary events, especially in the most severe patients.

## 1. Introduction

Irreversible airflow limitation represents the defining characteristic of chronic obstructive pulmonary disease (COPD) [1]. However, this functional abnormality is no longer regarded as the sole component of the disease, and COPD is now considered a complex condition that involves several manifestations, both pulmonary and extrapulmonary, besides airflow limitation. In this case, “complex” means that these different elements display nonlinear interactions [2], and the final result has been considered a syndrome rather than a disease [3]. Lung function measurement is essential for diagnosis, and Forced Expiratory Volume in the 1st second (FEV1) has been used as the only parameter for COPD management for many years [4]. However, FEV1 is obviously not sufficient to describe such complexity. The 2011 version of the GOLD document has added for the first time symptoms and exacerbation history in patient classification, thus creating four groups, from A (milder symptoms and low risk, defined as FEV1 ≥ 50% predicted and no frequent/severe exacerbations) to D (more severe symptoms and high risk, with either FEV1 < 50%, a history of frequent/severe exacerbations or both) [5]. More recently, several tools, including a “COPD control panel”, endo-phenotyping, and treatable traits characterization have been proposed to better manage COPD patients according to precision medicine objectives [6]; nevertheless, a widely accepted approach to COPD complexity is lacking [7].

COPD is characterized by systemic inflammation, which likely plays a role in biologically linking pulmonary and extrapulmonary manifestations [8]; interestingly, low-grade, systemic inflammation is also a well-known risk factor for future cardiovascular events in the general population [9] through its pro-atherogenic action [10]. Among the several cytokines involved in systemic inflammation, interleukin-6 has a relevant role both in COPD and atherogenesis [8,9,10].

The endothelium represents the pivotal element in atherogenesis and cardiovascular diseases [11], but it is currently viewed also as an important player in COPD pathogenesis through several purported mechanisms (transendothelial leukocyte migration, cell apoptosis or senescence, endothelial dysfunction) [12]; moreover, endothelial injury represents a common feature between pulmonary and systemic manifestations in COPD [13].

Cell-derived extracellular vesicles (EV) are small vesicles, released by virtually all eukaryotic cells into the bloodstream, where they play pleiotropic roles in intercellular communication, both in pulmonary and cardiovascular diseases [14,15], through several mechanisms, including microRNA [16] and mitochondria cargo [17]. Moreover, circulating EV (in particular endothelial-derived and monocyte-derived EV—EEV and MEV, respectively) have been implied in conditions characterized by systemic inflammation, such as chronic respiratory and cardiovascular diseases [18,19]. Lastly, EEV have been recognized as markers of endothelial dysfunction [20].

Although a previous study found a significant, direct relationship between increasing plasmatic EEV levels and severity of airflow limitation [21], no studies have so far explored the value of EV in a multidimensional evaluation of COPD, or their possible relationships with systemic inflammation. Therefore, we conducted a pilot observational study to analyze circulating EEV and MEV levels in COPD patients grouped according to the 2011 GOLD classification, which incorporates three main domains (airflow limitation, symptoms, exacerbations) together in a single panel. Based on the result from both the aforementioned study [21] and other works suggesting a role for EV as biomarkers in COPD [14], we would expect increasing EV levels along with the progression of disease burden, from group A to D. Moreover, we evaluated the relationships between both types of EV (EEV and MEV) and markers of systemic inflammation in this population.

## 2. Materials and Methods

### 2.1. Study Design, Subjects, and Procedures

We enrolled patients with stable COPD diagnosed according to the 2011 GOLD document [5], with the following exclusion criteria: chronic respiratory failure; recent (within 6 weeks) COPD exacerbation, acute coronary syndrome or pulmonary embolism; history of asthma; active cancer. We recorded symptoms using both the modified Medical Research Council scale and the COPD Assessment Test (CAT) [5]; all patients performed complete pulmonary function tests (PFTs), according to current indications [22] and after a 24 h withdrawal of inhaled therapy.

On a subsequent day, patients underwent blood tests for common biochemistry markers of cardiovascular injury (N-terminal pro-brain natriuretic peptide—NT-proBNP—and troponin), which are commonly used circulating markers of systemic inflammation (white blood cells count, C reactive protein, fibrinogen, interleukin-6—IL-6—and tumor necrosis factor-α, TNF-α) [23] and EV enumeration. All blood samples have been drawn from an antecubital vein, and all patients were in fasting conditions; active smokers were asked to refrain from smoking the day of sample collection.

### 2.2. EV Characterization and Analysis of Cytokine Concentration

The following monoclonal antibodies were used: anti-CD62E (allophycocyanin labeled, Clone 68-5H11, Mouse IgG1 k, BD Bioscience, Franklin Lakes, NJ, USA), anti-CD31 (phycoerythrin labeled, Clone L133.1, Mouse IgG1, k, BD Bioscience), anti-CD14 (phycoerythrin-Cyanine7 labeled, Clone M5E2, Mouse IgG2a, κ BD Bioscience), Mouse IgG1, κ Isotype Control (phycoerythrin labeled, BD Bioscience), Mouse IgG1, κ Isotype Control (allophycocyanin labeled, clone MOPC-21, BD Bioscience), Mouse IgG2a, κ Isotype Control (allophycocyanin labeled, BD Bioscience), and Annexin V (Peridinin-Chlorophyll-Protein -Cy™5.5 labeled, Clone Annexin V, BD Bioscience).

For EV measurement, blood (4 mL) was drawn into tubes containing sodium citrate (0.38% *w*/*v* final concentration). Platelet-poor plasma (PPP) was obtained by two subsequent centrifugations: 1500× *g* for 15 min and 16,000× *g* for 5 min at 4 °C. PPP was stored at −80 °C until use. EV analysis was performed in 200 μL of PPP by multiparametric flow cytometry, as described by us and others [21,24], with some modifications. Briefly, prior to flow cytometry, the EV suspension is incubated in the dark for 30 min at room temperature with 5 μL of fluorescent-conjugated monoclonal antibodies against cell-type specific antigens or isotype-matched controls and 5 μL of Annexin V. Then, EV are discriminated by size, using calibration beads (Megamix, Stago, Milan, Italy), as events conforming to a light scatter distribution within the 0.5–0.9 μm range in an SSc vs. FSc window and further labeled with annexin V to identify medium-large EV expressing phosphatidylserine [25]. Positivity to CD31 and CD62E was used to identify EV of endothelial origin. CD14 positivity was used to identify EV of monocytic origin. Flow cytometry was performed on a FACS-CANTO^TM^ (Becton Dickinson, Franklin Lakes, NJ, USA). EV were numbered as events/min in a low flow setting.

Plasma concentrations of IL-6 and TNF-α were measured by a sandwich ELISA kit (Kit-Elisa-Ready-SET-Go!, Affimetrix, Santa Clara, CA, USA) with a microplate reader (iMark™ Microplate Absorbance Reader, Bio-Rad, Milan, Italy) according to the manufacturer’s instructions. Routine laboratory parameters were evaluated in venous blood samples collected according to standard laboratory techniques by the Pisa University Hospital clinical laboratory.

### 2.3. Statistical Analysis

Data are presented as median values and interquartile ranges [IR]; non-parametric Jonckheere–Terpstra tests for independent samples (with post-hoc pairwise test) or (when appropriate) Mann–Whitney tests were used for comparisons among groups; correlations were assessed using Spearman’s rank correlation coefficient. IBM SPSS^®^ 20.0 was used for statistical analysis. The study was approved by the local Ethic Committee in compliance with the Declaration of Helsinki; all participants signed a written informed consent.

## 3. Results

Thirty-five patients (male/female: 25/10; age (median (IR)) 71.0 [6.0] years) completed the aforementioned study protocol. They were all current or former smokers and were divided according to the 2011 GOLD document [5] as follows: group A = 4 subjects; group B = 8 subjects; group C = 9 subjects; group D = 14 subjects. Four more patients were excluded from the final analysis: two because of exacerbation occurrence, one for consent withdrawal, and one for inadequate EV sampling. All patients who completed the study were on regular inhalation treatment; that was not stopped (except a 24 h withdrawal before PFTs) nor modified. Table 1 reports the main anthropometric, functional, and clinical data of the study population. No statistically significant differences were observed among the four groups; we have not performed statistical analysis on FEV1 values, mMRC, and CAT scores, because they differ by definition among the four groups, since the 2011 GOLD document specifically divides COPD patients according to FEV1 and symptoms (mMRC and CAT) values [5].

In Table 2, we reported the results of cardiac and inflammatory markers in the four groups: we did not find any significant differences (including in routine blood biochemistry, data not shown) except for IL-6, which shows a significant trend for increasing levels from group A to D (*p* for trend <0.05, by Jonckheere–Terpstra test, Table 2).

**Figure 1:**
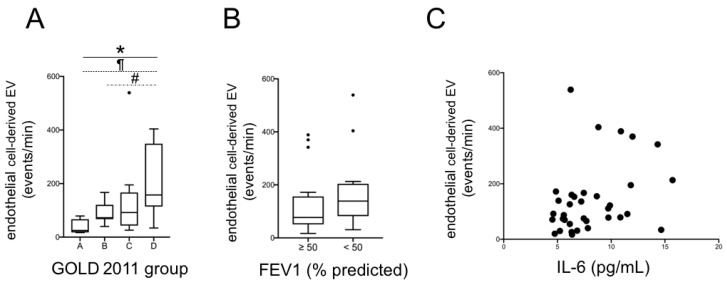
Total EEV according to 2011 GOLD group (**A**) and to FEV1 (**B**) (*: *p* < 0.001 for trend; ¶ and #: *p* < 0.05 for group D versus group A and B, respectively; Jonckheere–Terpstra test). Correlation between total EEV and plasma IL-6 (**C**). EEV: endothelial-derived extracellular vesicles; FEV1: Forced Expiratory Volume in the 1st second; IL-6: interleukin-6.

**Figure 2:**
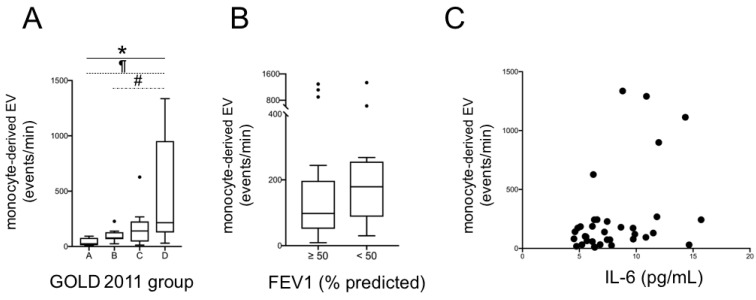
Total MEV according to 2011 GOLD group (**A**) and to FEV1 (**B**) (*: *p* < 0.001 for trend; ¶ and #: *p* < 0.05 for group D versus group A and B, respectively; Jonckheere–Terpstra test). Correlation between total MEV and plasma IL-6 (**C**). MEV: monocyte-derived extracellular vesicles; FEV1: Forced Expiratory Volume in the 1st second; IL-6: interleukin-6.

When analyzing EV values, we found a significant trend for increasing EEV levels, from group A to D (*p* for trend <0.001, by Jonckheere–Terpstra test); the post hoc pairwise analyses revealed significantly (*p* < 0.05) higher values in group D than in groups A and B (Figure 1A). We observed the same behavior for MEV with significantly increasing levels from group A to D (*p* for trend <0.001), and higher values in group D than in A and B (*p* < 0.05) (Figure 2A).

Then, we analyzed, by using the Mann–Whitney test, EEV and MEV levels after dividing patients in two groups, exclusively according to lung function, using a FEV1 cut-off value of 50% predicted (the same identified as clinically meaningful by the 2011 GOLD document [5]): no significant difference was found (Figure 1B and Figure 2B). Lastly, we found a significant correlation between EEV and IL-6 (rho = 0.35; *p* = 0.04; Figure 1C), while there was only a weak, not statistically significant trend between MEV and IL-6 (*p* = 0.07, Figure 2C).

We did not find any difference in EEV or MEV levels when the whole population was divided according to birth sex (not shown).

## 4. Discussion

To our knowledge, our results show for the first time a significant increase in circulating endothelial- and monocyte-derived extracellular vesicles (EEV and MEV, respectively) levels along with COPD progressive stages, defined by a multidimensional approach, including airflow limitation, symptoms, and exacerbations history [5]. Moreover, we found that IL-6, a well-known marker of systemic inflammation in COPD [23] and in atherogenesis [9], increases from group A to D and directly correlates with EEV levels.

We decided to divide patients according to the 2011 GOLD document, since it probably represents a reasonable compromise for COPD evaluation in clinical practice, with respect to both previous and subsequent versions. Indeed, until 2011, COPD management relied only on FEV1 values [4], but it is well known that FEV1 shows only a weak correlation with some important patient-centered outcomes, such as symptoms or exacerbations [26]. On the other hand, the current GOLD document suggests considering FEV1 measurement only for diagnosis, but its role in the further management of COPD has been downsized [1]; nevertheless, FEV1 measurement and its decline over time still represent relevant parameters in a comprehensive approach to COPD patients [27]. Actually, each of the three main domains simultaneously used by the 2011 GOLD document has been proposed to represent a specific feature of COPD: airflow obstruction expresses the severity, symptoms express the impact, and exacerbations represent the activity of disease [7]. Moreover, it has been demonstrated that the 2011 classification retains a significant prognostic value, since it captures the clinical relevance of symptoms and comorbidities (especially cardiovascular diseases) in these patients [28]. Anyway, the best way to approach COPD complexity is still debated [6].

In this scenario, EV can represent one of the possible tools helping clarify COPD complexity in order to shed light on its pathological processes as well as to provide a personalized approach to patients affected by this disease. Our data, showing an association between both EEV and MEV and disease burden, and a correlation between EEV and IL-6, are consistent with the systemic and complex nature of COPD. Specifically, the identical behavior of both EV and IL-6 levels from group A to D suggests the presence of a significant interplay between pulmonary disease and inflammation, with non-respiratory cells (in particular, endothelial cells and monocytes) involvement. In a previous study, some authors found significantly increasing EEV levels along with airflow obstruction severity in COPD patients [21]; we did not find this behavior in our population when analyzing EEV and MEV levels only according to FEV1 values; moreover, in the aforementioned study, no markers of inflammation were evaluated. Our results on the relationships between EEV and IL-6 are partly consistent with previously published data [29]; however, the cited study focused on exosomes, which are smaller vesicles, isolated and characterized with different approaches compared to EV, which represent the focus of our research; furthermore, the cellular origin of exosomes was not clearly specified by the authors. As already said, endothelium is now regarded as a main actor in COPD extrapulmonary manifestations [13]: thus, the EEV behavior we have observed in our population suggests a progressive endothelial involvement with increasing COPD severity, and the biological link is probably represented by inflammation and specifically by IL-6. Indeed, high circulating IL-6 levels are associated to cardiovascular risk in the general population [9,30], and the specific modulation of IL-6 signaling has already been proposed to reduce cardiovascular events in at-risk populations [31]. Moreover, IL-6 persistent elevation is associated with both a poor prognosis and an excess of cardiovascular events in a large population of COPD patients, such as the one recruited in the ECLIPSE (Evaluation of COPD Longitudinally to Identify Predictive Surrogate Endpoints) cohort [23].

The role of monocytes in persistent inflammation, both in COPD [32] and in atherosclerosis [15], is well known: increased levels of MEV have been detected in bronchoalveolar fluid of smokers with COPD, with respect to both smokers without COPD and non-smokers [33]. Moreover, in a study by Chiva-Blanch et al., MEV correlate with long-term prognosis after acute myocardial infarction, particularly for cardiovascular-specific mortality [34]. Therefore, the progressive increase in MEV we have found in our population (Figure 2A) can be viewed in this biological framework of systemic inflammation, linking pulmonary and extrapulmonary manifestations in COPD.

Of course, our study has some limits: first and foremost, the small sample size and the cross-sectional design do not allow general conclusions; furthermore, the patients’ distribution across the four groups is quite inhomogeneous, since group A is poorly represented, even though this can be expected, since the present study has been conducted in a university clinic for respiratory disease, and these less symptomatic patients are known to rarely seek specialist consult.

Since we have chosen a double labeling with CD31 (usually expressed on endothelial cells, including during the apoptotic process) and CD62E (expressed on “activated” EEV) [21], to be sure to identify endothelial-derived EV [35], we cannot be sure about the main origin of EEV (endothelial apoptosis or activation); however, the direct relationship we found between EEV and IL-6 suggests the release of EEV upon an inflammatory stimulus. Moreover, we found that among the inflammatory markers, only IL-6 shows significant differences across the four groups. We might have expected a similar increase in other markers as CRP, which is known to be biologically linked to IL-6. However, previous longitudinal studies on large cohorts [23] showed that a stable association of two or more inflammatory markers is present only in a minority (less than 20%) of COPD patients.

Lastly, we know that flow cytometry does not recognize smaller vesicles, since the laser beam of the flow cytometer does not resolve light scattered by particles smaller than 300 nm [36], although no other method has been proven ideal for EV detection [37], and this approach has been successfully used in previous similar studies [21].

## 5. Conclusions

In conclusion, we found that circulating endothelial- and monocyte-derived extracellular vesicles increase along with COPD severity, which was defined by using a multidimensional system that is probably more suitable to capture the disease complexity; furthermore, the relationship among EEV and IL-6 suggests a biological link between inflammation and endothelial activation/damage.

Since both IL-6 and endothelial activation are thought to contribute to cardiovascular morbidity in COPD, EEV and MEV might help identify a high-risk population for future cardiovascular events, especially in the most severe patients. In this light, our data, though preliminary, represent a starting point for larger, longitudinal studies in the field.

## Figures and Tables

**Table 1:** Anthropometric and functional characteristics of patients as a whole and divided according to the 2011 GOLD document (median (interquartile range)).

	All (*n* = 35)	Group A (*n* = 4)	Group B (*n* = 8)	Group C (*n* = 9)	Group D (*n* = 14)	*p* (Jonckheere-Terpstra)
Age, yrs	71.0 (6.0)	74.5 (10.0)	70.0 (10.0)	67.0 (13.0)	71.0 (3.0)	n.s.
male/female	25/10	4/0	6/2	8/1	7/7	n.s.
Smoking status						n.s.
Current/former	8/27	1/3	4/4	0/9	3/11	
Pack-years	50.0 (27.0)	39.0 (60.0)	52.5 (16.8)	50.0 (56.5)	48.5 (38.8)	n.s.
Dyspnea, mMRC	1.0 (1.0)	1.0 (1.0)	1.5 (1.0)	1.0 (0.0)	2.0 (1.0)	n.a.
CAT	11.0 (8.0)	7.5 (2.5)	10.5 (4.5)	7.0 (8.5)	15.5 (9.8)	n.a.
BMI, Kg/m^2^	28.2 (8.3)	26.7 (10.3)	31.4 (13.5)	28.7 (6.8)	26.9 (7.3)	n.s.
FEV1, L	1.28 (0.67)	1.52 (0.59)	1.74 (0.63)	1.34 (0.46)	0.97 (0.45)	n.a.
% pred.	52.0 (17.0)	62.5 (30.0)	62.0 (12.3)	45.0 (18.5)	49.5 (16.5)	n.a.
FEV1/FVC %	47.0 (16.0)	52.0 (17.5)	52.5 (12.3)	39.0 (20.0)	45.0 (15.5)	n.s.
DLCO, mL/min*mmHg						
% pred	15.9 (8.2)	16.9 (9.7)	16.8 (7.8)	23.8 (9.2)	14.6 (8.6)	n.s.
	64.0 (29.0)	84.0 (59.5)	54.0 (27.3)	93.0 (43.5)	59.5 (16.0)	n.s.
Therapy						
LAMA	1			1		
LABA-LAMA	15	3	5	3	4	
LABA-ICS	3		1	1	1	
LAMA-LABA-ICS	15	1	2	3	9	
LABA	1			1		

n.s.: not significant; n.a.: not applicable. mMRC: modified Medical Research Council; CAT: COPD Assessment Test; BMI: Body Mass Index; FEV1: Forced Expiratory Volume in the 1st second; FVC: Forced Vital Capacity; DLCO: diffusing capacity of the lung for CO; LAMA: long-acting antimuscarinics; LABA: long-acting beta2-agonists; ICS: inhaled corticosteroids.

**Table 2:** Cardiac and inflammatory markers among the four groups (median (interquartile range)).

	Group A (*n* = 4)	Group B (*n* = 8)	Group C (*n* = 9)	Group D (*n* = 14)	*p* (Jonckheere-Terpstra)
NT-proBNP, pg/mL	173 (157)	132 (95)	49 (151)	82 (174)	n.s.
Troponin, ng/L	17 (9)	16 (13)	14 (15)	11 (9)	n.s.
White blood cells, 10^3^/mm^3^	7.81 (2,84)	7.18 (3.79)	6.27 (2.28)	6.10 (2.42)	n.s.
CRP, mg/dL	0.23 (0.18)	0.31 (0.64)	0.61 (0.77)	0.22 (0.14)	n.s.
Fibrinogen, mg/dL	354 (137)	362 (57)	327 (119)	373 (112)	n.s.
IL-6, pg/mL	5.80 (4.86)	7.33 (2.08)	6.23 (2.93)	9.81 (6.07)	0.01
TNF-α, pg/mL	21.21 (5.23)	32.27 (26.24)	30.88 (24.16)	30.59 (17.35)	n.s.

NT-proBNP: N-terminal pro-brain natriuretic peptide; CRP: C reactive protein; IL-6: interleukin-6; TNF-α: tumor necrosis factor-α; n.s.: not significant.

## Data Availability

The datasets generated and/or analyzed during the current study are available from the corresponding author on request.
